# The Transcript Profile of a Traditional Chinese Medicine, *Atractylodes lancea*, Revealing Its Sesquiterpenoid Biosynthesis of the Major Active Components

**DOI:** 10.1371/journal.pone.0151975

**Published:** 2016-03-18

**Authors:** Shakeel Ahmed, Chuansong Zhan, Yanyan Yang, Xuekui Wang, Tewu Yang, Zeying Zhao, Qiyun Zhang, Xiaohua Li, Xuebo Hu

**Affiliations:** 1 Department of Medicinal Plant, College of Plant Science and Technology, Huazhong Agricultural University, Wuhan, 430070, P.R. China; 2 Center for Plant Functional Components, Huazhong Agricultural University, Wuhan, 430070, P.R. China; 3 National-Regional Joint Engineering Research Center in Hubei for Medicinal Plant Breeding and Cultivation, Huazhong Agricultural University, Wuhan, 430070, P.R. China; 4 Engineering Research Center for Medicinal Plants, Huazhong Agricultural University, Wuhan, 430070, P.R. China; Key Laboratory of Horticultural Plant Biology (MOE), CHINA

## Abstract

*Atractylodes lancea* (Thunb.) DC., named “Cangzhu” in China, which belongs to the Asteraceae family. In some countries of Southeast Asia (China, Thailand, Korea, Japan etc.) its rhizome, commonly called rhizoma atractylodis, is used to treat many diseases as it contains a variety of sesquiterpenoids and other components of medicinal importance. Despite its medicinal value, the information of the sesquiterpenoid biosynthesis is largely unknown. In this study, we investigated the transcriptome analysis of different tissues of non-model plant *A*. *lancea* by using short read sequencing technology (Illumina). We found 62,352 high quality unigenes with an average sequence length of 913 bp in the transcripts of *A*. *Lancea*. Among these, 43,049 (69.04%), 30,264 (48.53%), 26,233 (42.07%), 17,881 (28.67%) and 29,057(46.60%) unigenes showed significant similarity (E-value<1e^-5^) to known proteins in Nr, KEGG, SWISS-PROT, GO, and COG databases, respectively. Of the total 62,352 unigenes, 43,049 (Nr Database) open reading frames were predicted. On the basis of different bioinformatics tools we identify all the enzymes that take part in the terpenoid biosynthesis as well as five different known sesquiterpenoids via cytosolic mevalonic acid (MVA) pathway and plastidal methylerythritol phosphate (MEP) pathways. In our study, 6, 864 Simple Sequence Repeats (SSRs) were also found as great potential markers in *A*. *lancea*. This transcriptomic resource of *A*. *lancea* provides a great contribution in advancement of research for this specific medicinal plant and more specifically for the gene mining of different classes of terpenoids and other chemical compounds that have medicinal as well as economic importance.

## Introduction

The plant *Atractylodes lancea* (Thunb.) DC., known as “Cangzhu” in China, “Khod-Kha-Mao” in Thailand [1] and its name in Japan is “So-ju-tsu”[1, 2]. *A*. *lancea* belongs to the Asteraceae family. The rhizome of *A*. *lancea*, generally called rhizoma atractylodes is used for treatment of influenza, rheumatic diseases, night blindness and a few digestive problems [3–5]. The history of using rhizomes of *A*. *lancea* as a drug can be traced back to Han dynasty (206BC-220AD), when it was described in Shen-nong-ben-cao-jing, the first Chinese pharmacopoeia. Later it was found that this herb include two species, *A*. *lancea* and *A*. *Chinensis* (DC.) Koids, known “Mao CangZhu” and “Bei CangZhu” separately in China and people have used these together as rhizoma Atractylodes [6].

**Fig 1:**
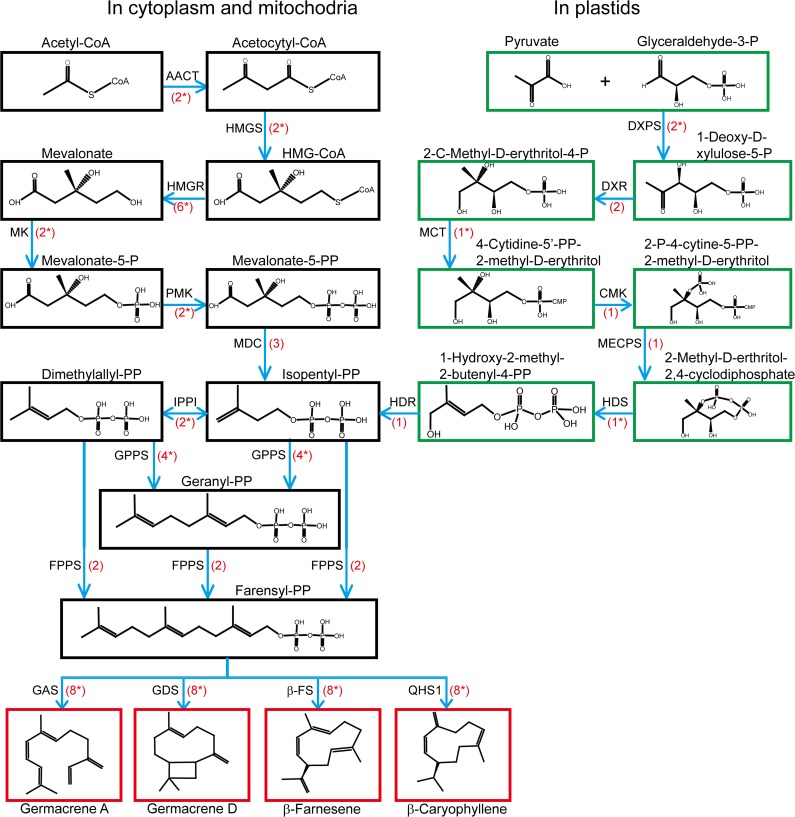
Putative sesquiterpenoid biosynthetic pathway in *Atractylodes lancea*. A flow diagram of biosynthetic pathway of terpenoid backbone and sesquiterpenoids biosynthesis in *Atractylodes lancea*. The structures of chemicals in the pathway are shown in boxes. The green boxes represent the plasticidal pathway while the black boxes show the pathway in cytoplasm & mitochondria. The words on the boxes are enzymes for the reaction while the numbers in red color represent the number of transcripts for that specific gene. Reactions in cytoplasm, mitochondria and plastids are shown in green. The boxes with red border show the structure of various sesquiterpenoids of *A*. *lancea*.

Previous reports imply terpenoids and their glycosidal derivatives are the major active components [7, 8]. Terpenoids are the natural products that are mostly present in plants with specific structures [9]. Plant-derived Terpenoids show a diversity of medicinal effects that comprise of multiple industrial and pharmaceutical applications including antiparasitic, anticancer, antifungal, antiviral and antibacterial activities. Terpenoids are grouped as monoterpenoids, sesquiterpenoids, diterpenoids, triterpenoids, and others [10]. In general, terpenoids are synthesized in plants by way of MVA pathway and MEP pathway. In the MVA pathway, terpenoid is synthesized starting from primary metabolic product acetyl-CoA to crucial precursors such as isopentyl diphosphate (IPP) and dimethylallyl diphosphate. The reaction is catalyzed by a large variety of enzymes with special product specificities (Fig 1). In the MEP pathway, glycolysis products glyceraldehydyde-3-phosphate and pyruvate are catalyzed into 1-deoxy-D-xylulose-5-phosphate. After a few enzymatic steps the pathway runs into the same chemicals as MVA pathway (Fig 1). The 1-deoxy-D-xylulose-5-phosphate synthase (DXS) and hydroxymethylglutaryl-CoA synthase (HMGR), the rate-limiting enzymes in MEP and MVA pathway, respectively, are usually encoded by a group of small multigene families [11]. Sesquiterpenes synthase are universally expressed family of different proteins which are able to convert the universal precursor farnesyl diphosphate (FPP) into more than three hundred various sesquiterpenes skeletons [12]. The current study mainly emphasises on the biosynthetic pathway of sesquiterpenoids in *A*. *lancea*.

Sesquiterpenoids have a wide variety of benefits such as pharmaceuticals, flavors, fragrances, industrial chemicals and nutraceuticals [13–15]. β-caryophyllene is an essential sesquiterpene that is present in different essential oils of many plants like cinnamon (*Cinnamomum cassia*), thyme (*Thymus mongolicus*), clove (*Syringa*spp.) and black pepper (*Piper nigrum*), of which mostly have been used for cure of different health problems as well as for fragrances [16, 17]. It is also prominently used as anti-carcinogenic & anti-microbial antioxidant, as well as skin penetration enhancer [18]. Germacrene D is another kind of sesquiterpene. It is a chiral compound, which is produced from FPP by enantionmers particular synthase [19]. Germacrene D has a sturdy effect on insect activities [20]. FPP can be converted into cyclic sesquiterpene, (E)-β-farnesene, which is catalyzed by (E)-β-farnesene synthase (β-FS)[21]. (E)-β-farnesene occurs in a variety of plants and animals & is widely used as a semio-chemical in insects and plants [22].

Sesiquiterpenes are the major components of the volatile essential oil from *A*. *Lancea*. In an effort to identify the chemical profiles of essential oil from *A*. *Lancea*, the wild grown plants produced mostly significant amount of sesiquiterpenes with the top three hinesol (68.5%), β-eudesmol (13.1%) and elemol (6.2%)[23]. However, the content of these chemicals is greatly influenced by the geographic location where the sample was taken from [23, 24], as *A*. *Lancea* is widely distributed in the vast area between Yellow River and Yangtze River of China. Among these diverse sesiquiterpenes, atractylenolides (I, II & III), atractylon, biatractylolide screened from *A*. *Lancea* were demonstrated to having a good protection against ethanol-induced gastric ulcer [25]. Atractylenolides were also proved to be insect repellents [26]. Recently it was found that a new sesquiterpenoid, hinesol, was responsible for the apoptosis in human cancer cells. On the contrary, the activity of β-eudesmol, a more commonly found sesiquiterpene in other medicinal plants also available from *A*. *Lancea*, was less effective as compared to hinesol [27]. There are other kinds of sesquiterpenes as guaiane, eudesmane, tricyclic carbon skeleton types, but the physiological activity remained to be elucidated [28]. Furthermore, it was proved that some other chemicals from *A*. *Lancea*, like atractylochromene, methylphenol derivatives, cyclohexadiene derivatives, polyacetylenes, atractylodin and acidic polysaccharides, showed diverse activities against inflammation, bacterial, fungi or obesity, but their structures are different from sesquiterpenes [29–31]. The chemical and structural diversity in *A*. *Lancea* correlates with its multiplex medicinal functions. Owing to recent advances in molecular biology and decreasing cost of next generation sequencing technology, RNA sequencing (RNA-seq) become a popular choice for the transcriptome studies especially in non-model species [32]. Consequently, RNA-seq has been extensively deployed in various TCM species, for example Chinese sage (*Salvia miltiorrhiza*)[33], Chinese Ginseng (*Panax ginseng*)[34] and Sanchi (*Panax notoginseng*). [35]. Deep transcriptome analysis also helps to discover various genetic profile, including alternative splicing isoforms [36], strand-specific expression [37] and microRNA discovery [38]. With the help of transcriptome sequencing, comprehensive information can be obtained on gene expression, molecular mechanisms and biological pathways, even in the absence of reference genome [39–43]. However, to date the study of *A*. *lancea* transcriptome is not reported yet. Here we report on the Illumina transcriptome sequencing, functional annotation and differential expression profiles in different tissues i.e. stem root, and leaf of *A*. *lancea* which will be an important resource for gene mining, genetic improvement and development of different molecular markers. Additionally, to further explore the differences of candidate unigenes in terpenoid biosynthesis among these *A*. *lancea* tissues, the transcriptional levels of all the related unigenes were concretely discussed. The results from our work could contribute to the discovery of genes dedicated to the terpenoid pathway and its accumulative regulation of volatile constituents in specific tissues of *A*. *lancea*. According to our information, the current research work is first report of secondary metabolic analysis in *A*. *lancea* based on *de novo* transcriptome analysis.

## Materials and Methods

### Collection of the *A*. *lancea* tissues

Prior to the experiment, the Institute of Science & Technology Development of HZAU university assured us that no specific permission is needed for the field experiment with *A*. *Lancea* in Hubei Province as it is commonly planted in China as a medicine sources. With the permission from Hubei Jintuyuan Forest Medicine & Seed Co. Ltd. (52 Jinyuanbao Avenue, Yuanbao, Lichuan City, Enshi autonomous district, Hubei province of China), experimental materials of *A*. *Lancea* was taken from a herbal medicine planting field (E08°56′, N30°18′) belongs to the company. The roots, stems and leaves of *A*. *lancea* were immediately frozen in liquid nitrogen after collection until use. The *A*. *lancea* was authenticated by Prof. Xuebo Hu, Assoc. Prof. Tewu Yang and Xuekui Wang.

### cDNA library preparation and sequence data analysis and assembly

To extract the total RNA present, equivalent weight of three tissue samples were mixed by using RNeasy Plant Mini Kits (Qiagen, Inc., Valencia, CA, USA) according to the manufacturer's protocol. All the samples of extracted RNA were qualified and quantified using a Nanodrop ND-1000 Spectrophotometer (Nanodrop Technologies, Wilmington, DE, USA), they showed a 260/280 nm ratio from 1.9 to 2.1. No sign of degradation was found when RNA samples were analyzed by electrophoresis. Transcriptome analysis was done by taking equal amounts of all the three samples by using Illumina's kit following manufacturer's protocol. Briefly, the poly-(A) mRNA was purified from the total RNA by Oligotex mRNA Mini Kit (Qiagen, Inc., Valencia, CA, USA) following the manufacturer's protocol. The cDNA library construction and normalization were performed using protocols described previously [44].

### Transcriptome *de novo* assembly

Trinity, a short read assembly package after sequencing was used for assembling Transcriptome mechanism, which consists of Inch-worm, a huge amount of RNA-seq reads were generated when processed sequentially by Chrysalis and Butterfly programs [45]. Consequent analysis of clean reads was carried out once they were filtered from the raw reads. Inchworms were the first to be used to assemble short reads with over-lapping sequences having longest contigs without gaps. Each cluster was used to construct a full de Bruijn graph after the clusters were grouped. Reads and pairs of reads were compared in equivalence to outline the pathways they had common. On the other hand full length transcripts were spliced isoforms, matching to paralogous genes, were generated by splicing apart transcripts. All such sequences from Trinity were defined as unigenes. In this study three samples of *A*. *lancea* were sequenced, sequence splicing was carried out for unigenes from each sample. Excess unigenes are separated from the required unigenes by using sequence clustering software. Unigenes are grouped into two classes after clustering genes into families: clusters (prefixed by CL) and single-tons (prefixed by unigene). Finally, we carried out alignment via BLASTx (E.value p 0.00001) between unigenes and protein databases of NR, Swiss-Prot, KEGG, and COG, and the course of unigene sequence was by using the best aligned results. If there is an incongruity among various databases, a priority order of NR, Swiss-Prot, KEGG, and COG was used to check the direction of the sequence. The unigenes whose sequences could not be determined by the above data base were aligned and their sequence directions determined using ESTScan [46].

### Unigene differential expression analysis

Differential expression of gene function was performed using gene ontology (GO) functional analysis, and these differentially expressed genes were mapped in each term using GO database (http://www.geneontology.org) and then correspondent number of gene with each GO term was determined. Following the creation of gene list which includes the number of genes linked with every GO term, the significance of GO enriched in differentially expressed gene in comparison with genomic background hyper-geometric test was applied.

### SSRs mining and primer design

SSRs consist of one to six nucleotide motifs, having minimum five tandem repeats. We used Microsatellite (MISA) detection tool for SSRs mining [47] and we design primer pairs using software primer3 (V.2.3.6) for each SSRs under default settings, with a range in the size of products of PCR from 100–250 bp [48, 49].

## Results and Discussion

### *A*. *lancea* transcriptome sequencing and unigene assembly

**Fig 2:**
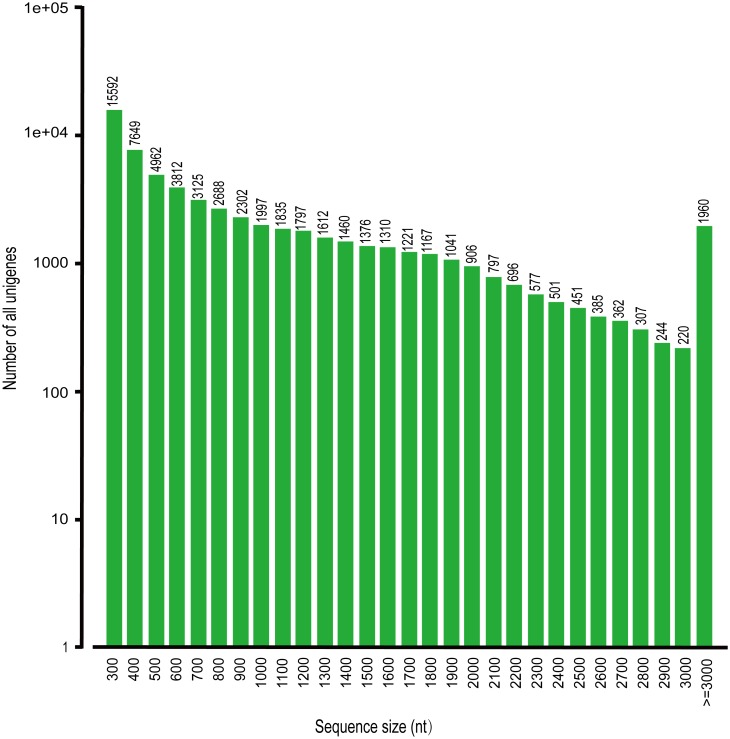
Length distribution of unigenes in *Atractylodes lancea*. The x-axis represent the size of the all assembled sequences and the y-axis indicates the corresponding number of unigenes.

To clarify a comprehensive overview of gene expression profiles in *A*. *lancea* tissues, the construction of cDNA libraries were made from different samples of leaf, root and stem of *A*. *lancea*, respectively and sequenced by the Illumina transcriptome platform in our experiments. After removal of adaptor sequences and low quality reads, a total of 43,921,277, 37,866,604 and 40,135,278 clean reads were acquired from leaf, root and stem tissues, respectively (Table 1). These data sizes are bigger than those from peanut (*Arachis hypogaea*) [44], yellow horn (*Xanthoceras sorbifolium*) [50], siberian apricot (*Prunus sibirica*) [51] and Centella (*Centella asiatica*) [52], suggesting that the relatively complete read databases were successfully constructed from different tissues of *A*. *lancea* by Illumina sequencing. Subsequently, Trinity software was used for assembly of these clean reads (Trinityrnaseq_r2013_08_14) and low density and quality reads were filtered out, resulting in 64,106, 55,409 and 56,565 unigenes in the leaves, root and stem respectively. After *de novo* assembly of three *A*. *lancea* tissues, 62,352 unigenes were finally obtained with an average length of 913 bp (Table 1). Among these, 42127 unigenes having a length range between 300 nt to 1000 nt and 15263 unigenes having a length longer than 1 kb (>1000 nt) as shown in Fig 2. Furthermore, we found that the sum of unigenes (62,352) in *A*. *lancea* is more than the identified number of unigenes 59,236 in peanut (*A*. *hypogaea*) [44], 51,867 unigenes in yellow horn (*X*. *sorbifolium*) [50] and 46,940 unigenes in Siberian apricot (*P*. *sibirica*) [51].

**Table 1:** Statistic of sequencing and de novo assembling of transcriptome in *Atractylodes lancea*.

	Sample	Total number	Total length(nt)	Mean Length(nt)	N50	Total consensus sequences	Distinct Clusters	Distinct Singletons
Contigs	Leaf	112883	42287508	375	806	0	0	0
	Root	94663	37505566	396	837	0	0	0
	Stem	101679	39492194	388	359	0	0	0
Unigenes	Leaf	64106	43921277	685	1258	64106	19718	44388
	Root	55409	37866604	684	1221	55409	16802	38607
	Stem	56565	40135278	710	1328	56565	16947	39618
Total		62352	56923290	913	1494	62352	23974	38378

### Functional annotation of *A*. *lancea* unigenes

**Fig 3:**
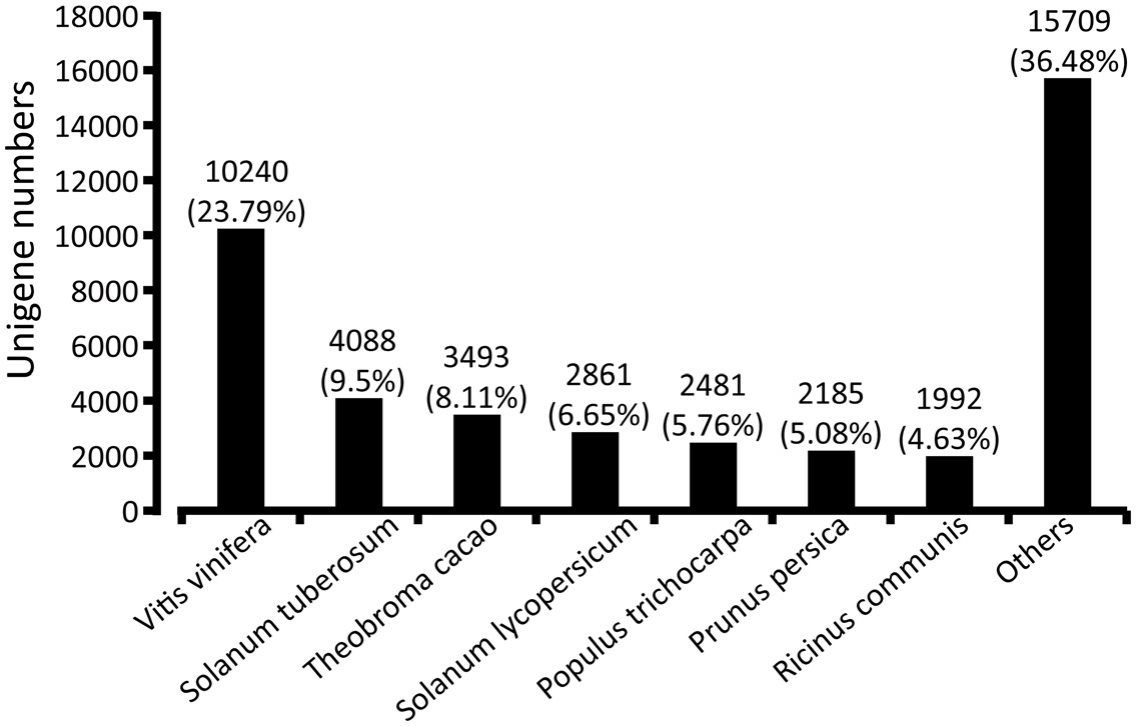
The species distribution of the non-redundant unigene annotation. The column shows the homology of *Atractylodes lancea* unigene number with that from other species. The numbers inside parentheses indicate the percentage of the homology to different species.

The species distribution of the non-redundant (Nr) annotation is shown in Fig 3. There was 23.79% of unigenes shown the highest homology to genes from grape (*Vitis vinifera*), 9.5% of unigenes matched to potato (*Solanum tuberosum*), 8.1% of unigenes matched to cacao (*Theobroma cacao*), 6.6% of unigenes matched to tomato (*Solanum lycopersicum*) and 5.8% & 5.1% of unigenes matched to populus (*Populus trichocarpa*) and peach (*Prunus persica*), respectively. All the *A*. *lancea* unigenes from different tissues were predicted via BLAST (basic local alignment search tool) with a cut-off E-value of 10^−5^ in public databases such as non-redundant (NR), SWISS-PROT, kyoto encyclopedia of genes and genomes (KEGG), classification of Orthologous Group (COG), and gene ontology (GO), which retrieved higher sequence similarity proteins among specific unigenes beside their functional annotations. From the BLAST results, a total of 43,049 (69.04%), 30,264 (48.53%), 26,233 (42.07%), 17,881(28.67%) and 29,057(46.60%) unigenes showed diverse similarity to well-known proteins in above mentioned databases, respectively (Table 2). However, 44,482 unigene (71.34%) sequence orientations are still unknown, which is higher than the peanut (*A*. *hypogaea*) (27.8%) [44] but lower than that of Chinese tulip tree (*Liriodendron chinense*) (73.60%) [53]. This is because of the lack of *A*. *lancea* genomic information, and few or no effective characterized protein domains of the shorter sequences for getting BLAST hits. Also, it is possible that some un-matched unigenes are the novel genes specific for *A*. *lancea*.

**Table 2:** Statistics of annotations for assembled unigenes of *Atractylodes lancea* in different public databases.

Database	Unigenes	Percentage(%)
NR	43049	69.04
SWISS-PROT	30264	48.53
KEGG	26233	42.07
COG	17881	28.67
GO	29057	46.6
ALL	44482	71.34

### Functional classification of *A*. *lancea* unigenes by GO, COG and KEGG

**Fig 4:**
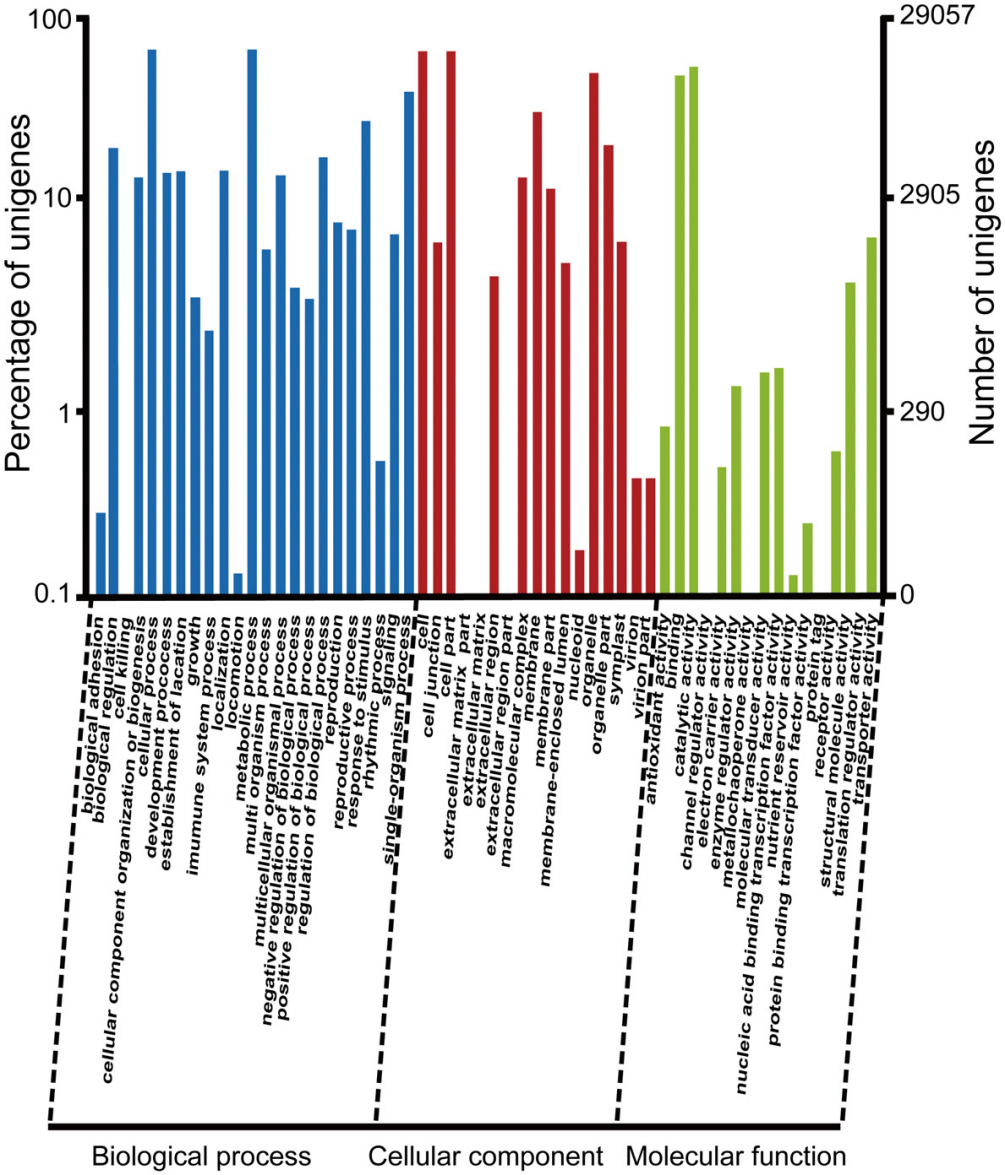
Distributions of GO annotation of all unigenes. The results were classified into three main categories: biological process, cellular component, and molecular function. The left y-axis indicates the percentage of a specific category of genes in that category. The right y-axis indicates the number of genes in a category.

For categorizing the function of predicted *A*. *lancea* unigenes gene, ontology (GO) annotation was used [54]. In total, 29,057 unigenes were selected for three main GO categories and 56 subcategories (Fig 4). It shows that “metabolic process”, “cellular process”, “binding” and “catalytic activity” are the most dominant category involving more than 180,000 unigenes, while a small portion of genes were linked with terms such as “pigmentation”, “receptor regulator activity” and “protein tag”. It is interesting to observe that 20,169 unigenes from GO analysis had not been annotated in the Swiss-Prot database, which could be explained by the fact that the prediction quality could be significantly improved by GO annotation as the clustering of proteins determine their sub cellular locations reflection in a better way [55].

**Fig 5:**
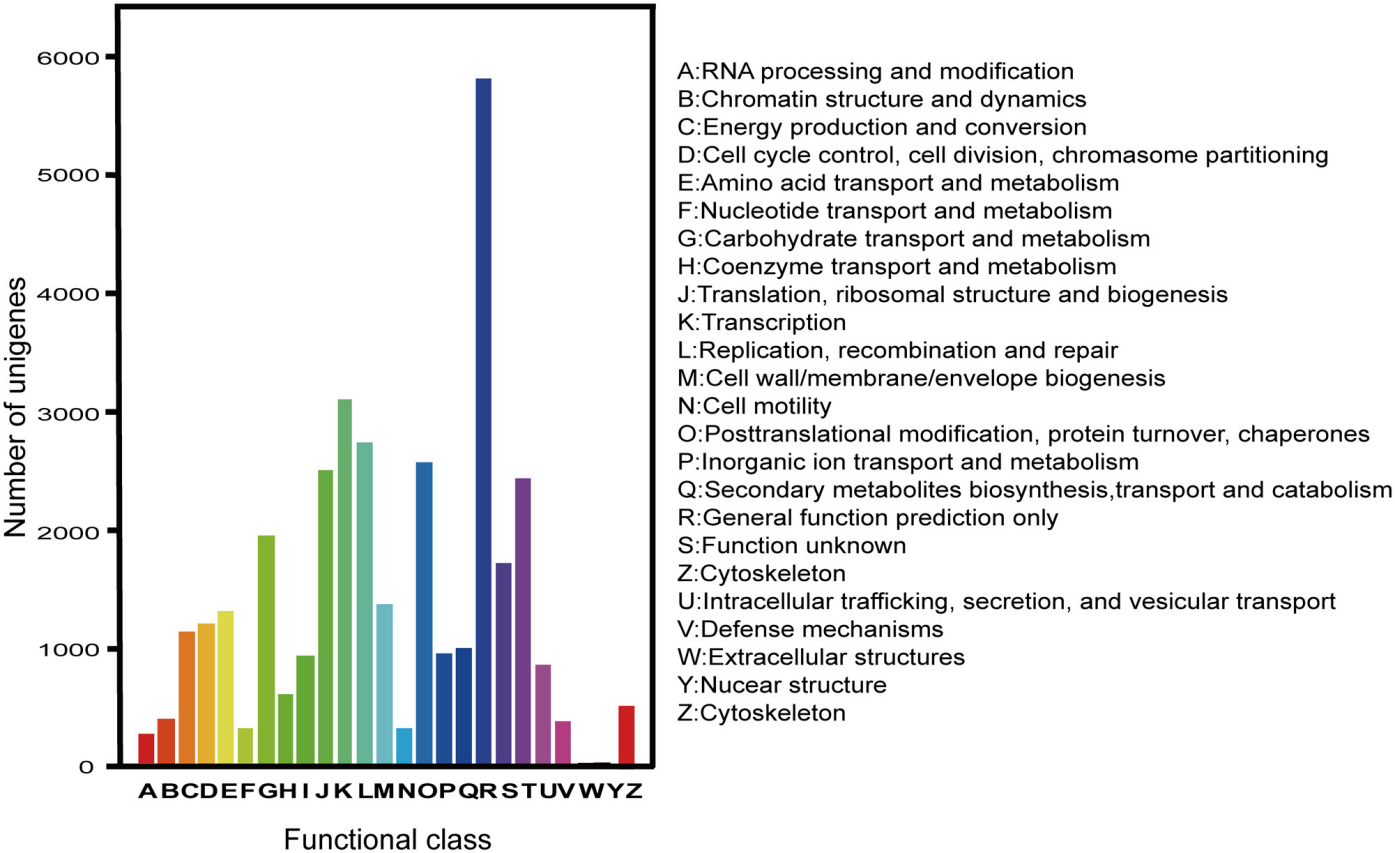
COG function classification of all unigenes. The annotated unigenes are divided into a variety of functional orthologous groups, which are indicated by letters A-Z and annotated besides the figure.

To further expose the value of annotation process and predict possible functions of unigenes, we looked for the annotated sequences for genes involved in the classification of orthologous group (COG) to classify the orthologous products of genes [56]. COG database was used for the alignment and for prediction and classification of possible function of all *A*. *lancea* unigenes. Results revealed that 17,881 unigenes were recognized as 25 COG classifications (Fig 5). In 25 COG categories, the largest group represents “general function prediction (5837 unigenes)”, second cluster was ‘transcription’ (3105 unigenes) and then ‘replication, recombination & repair’ (2732 unigenes). It was also observed that just a few genes found related to the terms as “extracellular structures” and “Nuclear structures".

For further recognition of the interaction and biological functions of genes in the *A*. *lancea*, KEGG was used to make canonical pathways as reference mapping of all annotated sequences [57]. KEEG was employed as a reference database of pathway networks for integration and interpretation of large scale datasets generated by high-throughput sequencing technology [58, 59]. On the fact that some unigenes were recruited in several KEGG pathways during the analysis, 26,233 unigenes were assigned to 128 KEGG pathways (S1 Table), of which most represented by Metabolic Pathway (5971 unigenes, 22.76% of annotated to KEGG database), followed by “biosynthesis of secondary metabolites” (2957 unigenes, 11.27% of annotated to KEGG database), “plant-pathogen interactions” (1608 unigenes, 6.13% of annotated to KEGG database), “Plant hormone signal transduction” (1396 unigenes, 5.32%of annotated to KEGG database) and “Ribosome” (1174 unigenes, 4.48% of annotated to KEGG database).

### Differentially expressed genes (DEGs) in the leaf vs. stem, leaf vs. root and root vs. stem in *A*. *lancea*

**Fig 6:**
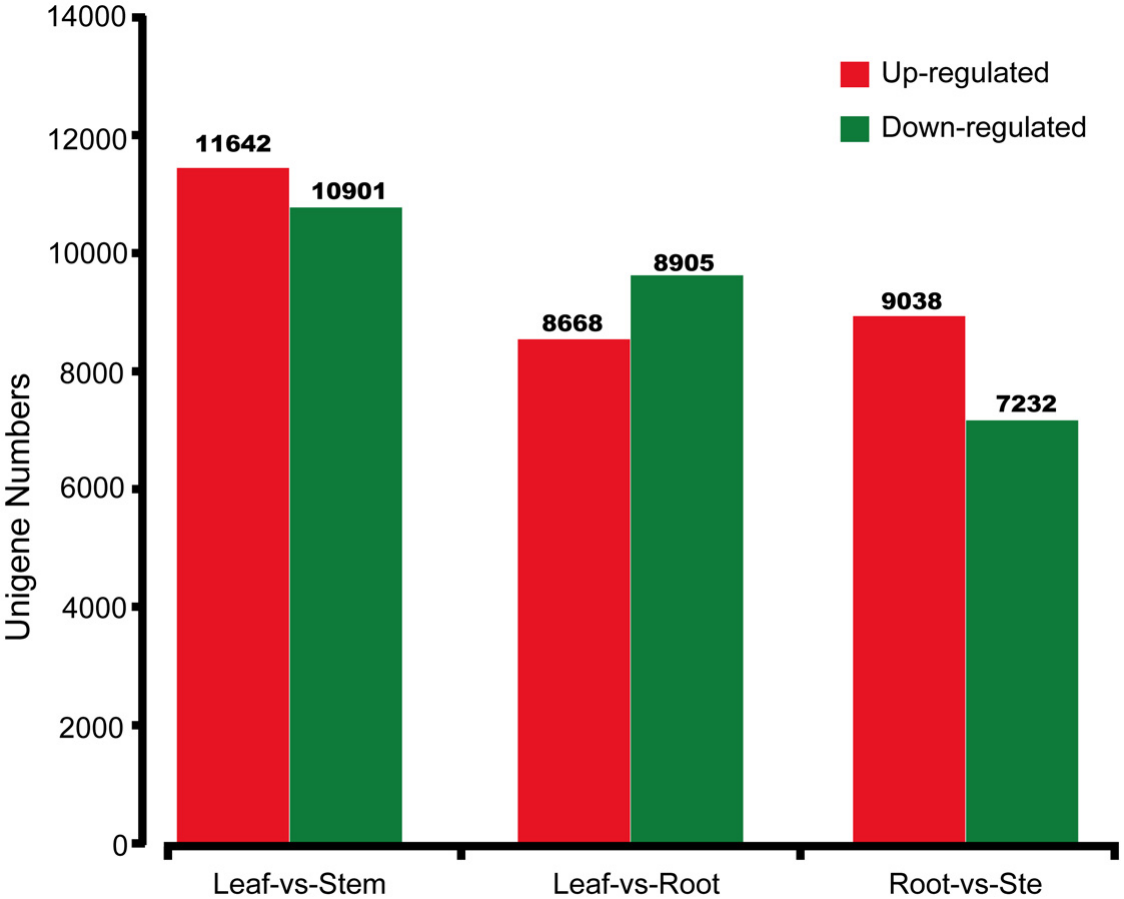
Differentially expressed genes profiling of three libraries of leaf, root and stem of *Atractylodes lancea*. The red and green columns indicate up- and down-regulated genes in comparisons of leaves, stem and root libraries in *A*. *lancea*. FDR≤0.05 and the absolute value of Log2FC Ratio ≥1 were used as the threshold to judge the significance of gene expression difference from transcriptome data.

A major function of the transcriptome sequencing is for comparison of levels of gene expression among different samples. To check the differences in expression of gene among three libraries from the leaf, stem and root, the tag frequencies of leaf vs. stem, leaf vs. root and root vs. stem were used. Through FPKM method (fragments per kb per million reads) all-unigene expressions were calculated. Firstly fragments density measures was normalized and for judgment of significance of gene expression false discovery rate(FDR) < 0.001 were used and the total value of |log2Ratio| ≥ 1 was used as a threshold. In Fig 6 the result shows a two-fold transcript difference among three libraries. We identified 22543, 18263 and 16370 unigenes in leaf vs. stem, leaf vs. root and root vs. stem libraries respectively that were differently expressed in all three libraries (S2 Table). Of these 11642, 8668 and 9038 unigenes were up-regulated and 10901, 9605 and 7232 unigenes in three libraries were down-regulated regulated by the log2 ratio bigger than 2 or less than 0.5 of leaf vs. stem, leaf vs. root and root vs. stem, respectively. It also showed among these differential expression genes, most were found expressed in the root, and then the stem and leaf. One assumption is that the diverse chemical synthesis of the plant is largely processed in the root.

### Analysis of *A*. *lancea* unigenes related to terpenoid backbonebiosynthesis

Based on the Nr annotation, a total of 77 Contigs/unigenes were identified as the genes of MVA pathway, that include acetyl CoA C-acetyltransferase (AACT), 3-hydroxy-3-methylglutaryl CoA synthase (HMGS), 3-hydroxy-3-methylglutaryl CoA reductase (HMGR), mevalonate kinase (MK), phosphomevalonate kinase (PMK), mevalonate-5-pyrophosphate decarboxylase (MDC), isopentenyl diphosphate isomerase (IPPI), geranyl diphosphate synthase (GPPS), farnesyl diphosphate synthase (FPPS), beta-caryophyllene synthase (QHS1), germacrene D synthase (GDS), germacrene A synthase (GAS) and E-β-farnesene synthase (β-FS). These genes produce β-caryophyllene, germacrene D, germacrene A and E-β-farnesene four different types of sesquiterpenoids [21, 60–62]. It also has to be pointed out that due to the limitation of short reads of RNA-seq, some unigenes assembled by the software are too short to represent real transcripts. Other unigenes are long enough to cover one or two domains of usual protein size, but they are almost identical to a longer transcript except a small part of the fragments. These unigenes are likely from one gene, possibly generated with selective transcripts or assembly error. In the end, we predicted 33 unigenes that are responsible for the enzymatic synthesis of MVA pathway (Fig 1). Nevertheless, these unigenes need to be approved by future cloning. Based on our analysis, up to 10 non-redundant unigenes were present in the plastidal MEP pathway, liable for the synthesis of the isopentenyl diphosphate that is the building block of terpenoids. These included 2 unigenes for 1-deoxy-D-xylulose-5-phosphate synthase (DXPS), two unigenes for 1-deoxy-D-xylulose-5-phosphate reductoisomerase (DXR), 1 unigene for 2-C-methyl-D-erythritol 4-phosphate cytidylyl transferase (MCT), 2 unigenes for 4-(cytidine 5'-diphospho)-2-C-methyl-D-erythritol kinase (CMK), 1 unigene for 2-C-methyl-D-erythritol-2, 4-cyclodiphosphate synthase (MECPS), 1 unigenes for 4-hydroxy-3-methyl but-2-(E)-enyl diphosphate (HDS), and 1 unigene for 4-hydroxy-3-methyl but-2-(E)-enyl diphosphate reductase (HDR). It is shown that most of candidate genes from the MEP pathway were up-regulated in leaves except DXPS and DXR. One DXPS (CL8765.Contig3_All) is up-regulated in the root and one (Unigene15742_All) is down-regulated in the stem. While in case of HDR, out of two unigene, (CL5530.Contig1_All) is up-regulated in the root (S1 Table). We also found that the genes from MEP pathway showed higher expression in leaves than in root and stem at the transcriptional level. Only one unigene was found codifying the isopentenyl diphosphate delta-isomerase which catalyzes the alteration of isopentenyl diphosphate Dimethylallyl diphosphate. Moreover, we found that prenyl-transferases, which generates higher-order building blocks: farnesyl diphosphate synthase (2 contigs/ unigene) and geranyl diphosphate synthase (8 unigenes), are the originator of different categories of terpenoids. The protein sequences of all the transcripts are provided in S1 Table.

### Analysis of *A*. *lancea* unigenes related to sesquiterpenoids biosynthesis

Sesquiterpenoids are derived from FPP which can be cyclized to produce various structures by different types of enzymes [63]. In this study, 19 contigs/unigenes (≥200 bp) were annotated to be involved in four different types of sesquiterpenoid biosynthesis, which includes β-caryophyllene synthase, germacrene D synthase, E-β-farnesene synthase and germacrene A synthase (Fig 1). These contigs/unigenes are CL4471.contig1_ALL, CL8689.contig1_ALL, Unigene14966_All, Unigene20711_All, Unigene20756_All, Unigene20757_All Unigene21327_All, Unigene21328_All, Unigene21329_All, Unigene21417_All, Unigene23621_All, Unigene25222_All, Unigene32673_All, Unigene33174_All, Unigene33794_All, Unigene34375_All, CL1332.Contig1_All, CL1748.Contig6_All and CL5528.Contig1_All. We noticed that, the above four sesquiterpenoid synthases share a high homology and it is difficult to separate them from each other without experimental confirmation. Furthermore, these enzymes are also homologous to sesquiterpene cyclase, β-pinene synthase, α-isocomene synthase, etc. All these enzymes are commonly grouped as sesquiterpene synthases. However, only one potential unigene (CL5528.Contig1_All) among the 19 sesquiterpene synthases is predicted to be germacrene A synthase with certainty. In another case, β-eudesmol synthase was reported for the specific sesquiterpene β-eudesmol biosynthesis [64]. But we were unable to find any candidate could match the enzyme with the current searching criteria. Previous study showed that βeudesmol was not always present in *A*. *lancea* samples [23, 24], In our study β-eudesmol synthase was not detected either because the gene expression was too low to be captured or because of the poor fragmentation or enrichment during the process of RNA-Seq.

It is very intriguing to pinpoint all enzymes especially for *A*. *lancea* sesquiterpenoid biosynthesis. Previous study indicates that cytochrome P450 oxidase (CYP) plays an important role in generation of all kinds of terpenoid derivatives [65]. In a search of all possible CYPs encoded by the *A*. *lancea* transcriptome, a total of 3,241 CYP contigs were found in the Nr annotation. Further filtration of redundant contigs with possibly the same predicted functions, the CYP quantity was narrowed down to 369 (S1 Table), however, it is still 1.5 times more than that of Arabidopsis thaliana [65], indicating a more sophisticated chemical process and diversity. Besides the sesquiterpene, there are other kinds of terpenes with less content in the *A*. *Lancea*. It correlates with the discovery of a large number of CYP genes, of which some are predicted to be terpene modifiers. All these components lay the foundation of chemical diversity for the fact that it treats various diseases.

The study on the biochemical properties of enzymes for sesiquiterpenes biosynthesis has made substantial progress in the past years, such as discovery of committed enzymatic steps in the biosynthesis of sesiquiterpenes [66, 67]. However, the identification and cloning of these enzymes are more challenging. Other than β-caryophyllene synthase, germacrene D synthase, E-β-farnesene synthase and germacrene A synthase, there are a few similar genes have been elucidated like tomato sesquiterpene synthase (Sst1) and Sst2 [68], aoeghum terpene synthase (SbTPS1-SbTPS7) [69], and a Cstps1, a sesquiterpene synthase-encoding genes for citurs aroma formation [70]. In our annotation database, we could sort out a bunch of sesquiterpene synthases. But due to the structural similarity between the sesquiterpenes, the sesquiterpene synthases also come with a close homology. Future study may explain whether those sesquiterpene synthase candidates can be grouped into further subgroups of each with the specificity to one kind of sesquiterpene.

### SSR markers development in *Atractylodes lancea*

**Fig 7:**
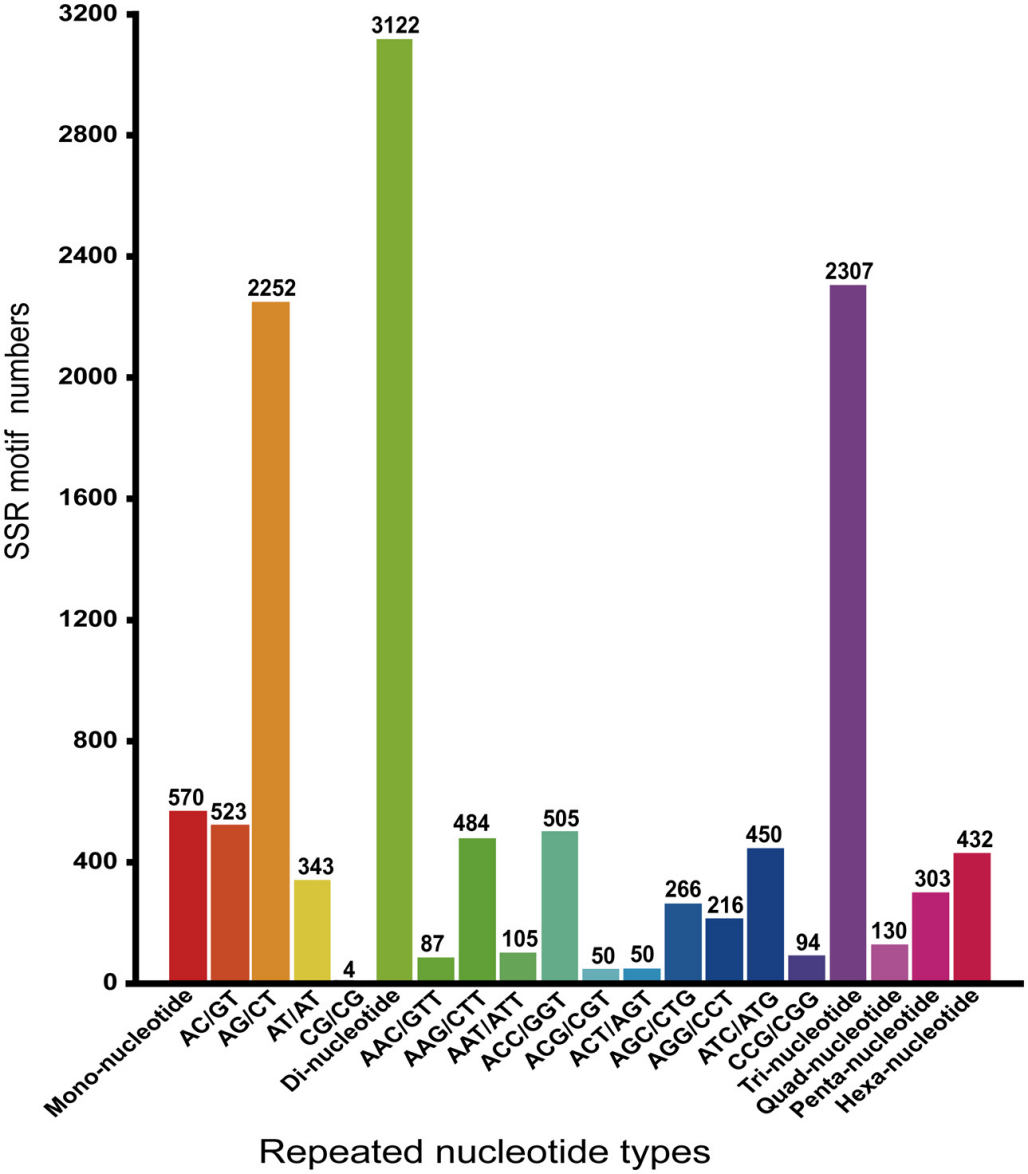
Quantity statistics of SSR classification: The X-axis is the repeat times of repeat units; the Y-axis is the number of SSRs from *Atractylodes lancea*. The di-nucleotide category was found in large number and among the di-nucleotide (AG/CT) was most abundant one in our SSRs.

SSRs are used as chief molecular markers. These repetitive DNA sequences symbolize a vital section of an advanced eukaryote genome. These typically co ascendant and highly polymorphic are widely utilized for marker systems of genetic mapping, molecular breeding in a wide variety of species [71–76]. In order to develop SSR markers in *A*. *lancea* and find potential microsatellites, all the 62,352 unigenes produced in current study were utilized, for all motifs they were defined as bi-hexa nucleotide SSR with at least four repeating units (except for di-nucleotide with a minimum of six repeating units, and tri-nucleotide with a minimum of five repeats). By using different primers (S1 Table), total of 6,864 microsatellites were identified in 5,970 unigenes, 757 unigenes contained more than 1 SSR. Di-nucleotide motifs were found to be the most abundant types (3,122, 45.48%), followed by tri-nucleotide (2,307, 33.61%), hexa-nucleotide (432, 6.29%), penta-nucleotide (303, 4.41%) and tetra-nucleotide (130, 1.89%), (Table 3). In our current study, *AG/CT* repeat was found the most abundant motif among all the searched SSRs, (2252, 32.80%), followed by *AC/GT* (523, 7.61%), *ACC/GGT* (505, 7.35%), and *AAG/CTT* (484, 7.05%) (Fig 7). Conventional methods for SSR marker development are expensive, arduous and time-consuming. The newly discovered and developed high-throughput sequencing technique is a powerful and cheap tool for transcriptome sequencing [77]. For microsatellite mining, SSR markers are being developed by the transcriptome data, and had been utilized in many species [55, 78, 79].

**Table 3:** A summary of SSRs identified in *Atractylodes lancea*.

Searching Items	Numbers
Total number of sequence examined	62352
Total size of examined sequence	56,9232,90
Total number of identified cSSRs	6864
Number of cSSRs containing sequences	5970
Number of sequences containing more than one cSSRs	757
Number of cSSRs present in compound formation	303
Mono-nucleotides	570
Di-nucleotides	3122
Tri-nucleotides	2307
Tetra-nucleotides	130
Penta-nucleotides	303
Hexa-nucleotides	432

### Discovery of simple nucleotide polymorphisms (SNPs)

**Fig 8:**
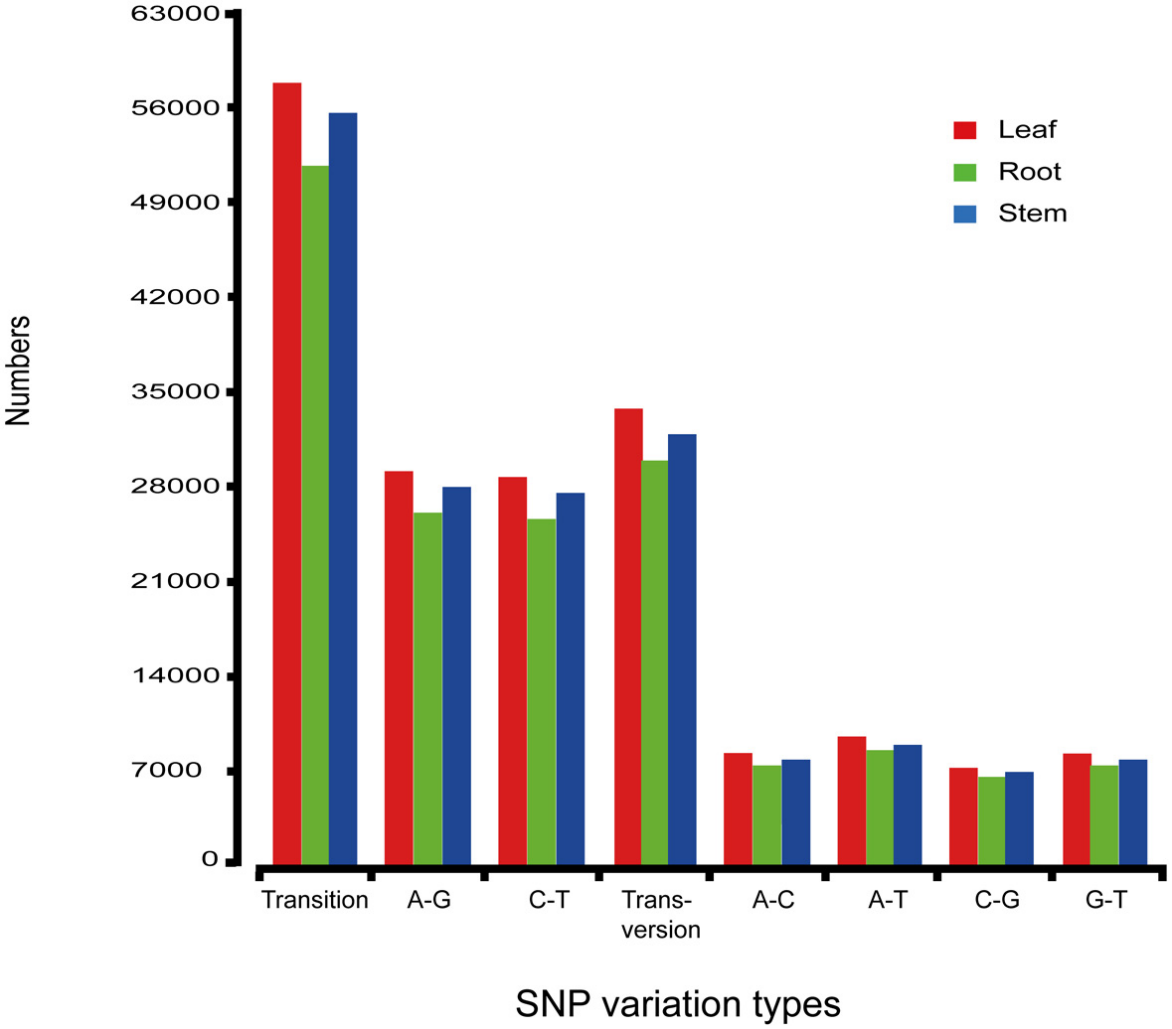
Statistics of SNP number. The X-axis is SNP types; the Y-axis is the number of SNP.

Innovating SNPs from cDNA libraries mapping revealed 91,540; 81,678 and 87,329 SNPs across 112,883; 94,663 and 101,679 contigs in leaves, root and stem of *A*. *lancea*, respectively (Table 4). A total of 260,547 heterozygous SNPs were detected from all three samples and out of these 165,120 were transitions and 95,427 were transversions (Fig 8). We also found several prospective SNP markers, which can be beneficial for the phylogenetic and population genetic studies of *A*. *lancea*. The identified SNP markers can be constructive to assist in genetic mark of selection for genetic association analysis in further research and also for identification of functional variations [80, 81]. The identification of huge SNPs provides affluence of potential markers to be helpful in various applications, such as linkage mapping, population genetics, and gene-based association studies and comparative genomics.

**Table 4:** A summary of SNP results in *Atractylodes lancea*.

SNP Type	Leave	Root	Stem	Total
Transition	57839	51702	55579	1,65,120
AG	29135	26042	28015	83192
CT	28704	25660	27564	81928
Transversions	33701	29976	31750	95427
AC	8428	7402	7902	23732
AT	9601	8564	8989	27154
GC	7281	6629	6952	20862
GT	8391	7381	7907	23679
Total	91540	81678	87329	2,60,547

## Conclusion

People in South-East Asian countries like China, Japan and Thailand make use of *A*. *lancea* as a traditional medicine for different diseases for a long time. Here we report the Illumina transcriptome sequencing, functional annotation and differential expression profiles in the different tissues i.e. stem root, and leaf of *A*. *lancea* which will be an important resource for gene mining, genetic improvement and development of different molecular markers. In current study 62,352 high quality unigenes were obtained from these tissues. Additionally we found the unigenes that are responsible for encoding the different enzymes that are involved it the biosynthesis of terpenoid backbone pathway as well as sesquiterpenoids which will help future functional & comparative genomic research on this important plant.

## Supporting Information

S1 Table Unigenes from the transcriptome of *Atractylodes lancea* by KEGG. 26,233 unigenes were assigned to 128 KEGG pathways.
(XLSX)

S2 Table Differentially expressed unigenes from three libraries of *Atractylodes lancea* showing up and down regulated unigenes (leaf vs stem), (leaf vs root), (root vs stem). (XLSX)

S3 Table Putative unigenes along their enzymes of the terpenoid backbone biosynthesis as well as four different types of sesquiterpenoids with their FPKM value showing their expression in three different tissues of *Atractylodes lancea*. (XLSX)

S4 Table Protein sequences of all the transcripts involved in the sesquiterpenoid biosynthesis. (TXT)

S5 Table All contigs and unigenes for CYP450 in *Atractylodes lancea*. (XLSX)

S6 Table Primers used for SSR analysis in *Atractylodes lancea*. (XLS)
